# A possible new syndrome with growth-hormone secreting pituitary adenoma, colonic polyposis, lipomatosis, lentigines and renal carcinoma in association with familial testicular germ cell malignancy: A case report

**DOI:** 10.1186/1752-1947-1-9

**Published:** 2007-03-28

**Authors:** Phuong L Mai, Larissa Korde, Joan Kramer, June Peters, Christine M Mueller, Susan Pfeiffer, Constantine A Stratakis, Peter A Pinto, Gennady Bratslavsky, Maria Merino, Peter Choyke, W Marston Linehan, Mark H Greene

**Affiliations:** 1 Clinical Genetics Branch, Division of Cancer Epidemiology and Genetics, National Cancer Institute, National Institutes of Health, Bethesda, Maryland, USA; 2 Westat Incorporated, Rockville, Maryland, USA; 3 Section on Endocrinology & Genetics, Developmental Endocrinology Branch, National Institute of Child Health and Human Development, National Institutes of Health, Bethesda, Maryland, USA; 4 Urologic Oncology Branch, National Cancer Institute, National Institutes of Health, Bethesda, Maryland, USA; 5 Division of Laboratory and Pathology, National Cancer Institute, National Institutes of Health, Bethesda, Maryland, USA; 6 Molecular Imaging Program, National Cancer Institute, National Institutes of Health, Bethesda, Maryland, USA

## Abstract

**Background:**

Germ-cell testicular cancer has not been definitively linked to any known hereditary cancer susceptibility disorder. Familial testicular cancer in the presence of other findings in affected and unaffected family members might indicate a previously-unidentified hereditary cancer syndrome.

**Case presentation:**

The patient was diagnosed with a left testicular seminoma at age 28, and treated with left orchiectomy followed by adjuvant cobalt radiation. His family history is significant for testicular seminoma in his son, bladder cancer in his sister, and lipomatosis in his father. His evaluation as part of an etiologic study of familial testicular cancer revealed multiple colon polyps (adenomatous, hyperplastic, and hamartomatous) first found in his 50 s, multiple lipomas, multiple hyperpigmented skin lesions, left kidney cancer diagnosed at age 64, and a growth-hormone producing pituitary adenoma with associated acromegaly diagnosed at age 64. The patient underwent genetic testing for Cowden syndrome (*PTEN *gene), Carney complex (*PRKAR1A *gene), and multiple endocrine neoplasia syndrome type 1 *(MEN1 *gene); no deleterious mutations were identified.

**Discussion:**

The constellation of benign and malignant neoplasms in the context of this patient's familial testicular cancer raised the possibility that these might be manifestations of a known hereditary susceptibility cancer syndrome; however, genetic testing for the three syndromes that were most likely to explain these findings did not show any mutation. Alternatively, this family's phenotype might represent a novel neoplasm susceptibility disorder. This possibility cannot be evaluated definitively on the basis of a single case report; additional observations and studies are necessary to investigate this hypothesis further.

## Background

Testicular germ cell tumor (TGCT) is the most common malignancy in U.S. adolescents and young adult males ages 15–45. TGCT risk factors include family history, personal history of previously-diagnosed TGCT, cryptorchidism and other disorders of male urogenital differentiation [1].

Approximately 1–3% of individuals with TGCT report having ≥ one affected first-degree relative. The relative risk of TGCT is higher among siblings than among fathers or sons of affected individuals [1], suggesting the possibility of genetic heterogeneity and environmental influences. Segregation analysis suggested that genetic susceptibility is important in familial TGCT, with recent studies indicating that multiple genetic loci of lower penetrance acting in concert contribute to the familial aggregation of this malignancy [2]. Although TGCT has been implicated in a number of genetic disorders, it has not been definitively linked to any known hereditary cancer syndrome [3].

Finally, individuals with TGCT are observed to have increased risks of certain cancers, including pancreas, kidney, urinary bladder, thyroid, lymphatic tissue, leukemia, and connective tissues [4]. Although the excess risk of several of these second malignancies has been attributed to late complications of testicular cancer treatment, the possibility that TGCT is part of a familial syndrome which includes other types of cancer cannot be dismissed.

We established the Multidisciplinary Etiologic Study of Familial Testicular Cancer in 2002 (NCI Protocol 02-C-0178) to identify possible testicular cancer susceptibility genes and to characterize more precisely the clinical phenotype of the familial testicular cancer syndrome. During the course of this study, we encountered a family with seminoma in a father and son, and a provocative constellation of other cancers and benign conditions in the father. The following case report summarizes our attempt to explain these findings on the basis of a known cancer susceptibility disorder.

## Case report

The proband was a 63 year-old, Caucasian male who presented to the NCI Clinical Genetics Branch in October 2004 for evaluation of familial testicular cancer. He developed a left testicular pure seminoma at age 28 (per medical records; no pathology was available for review); he underwent a left radical orchiectomy followed by adjuvant cobalt irradiation to the retroperitoneum, and has remained free of testicular cancer.

**Figure 1:**
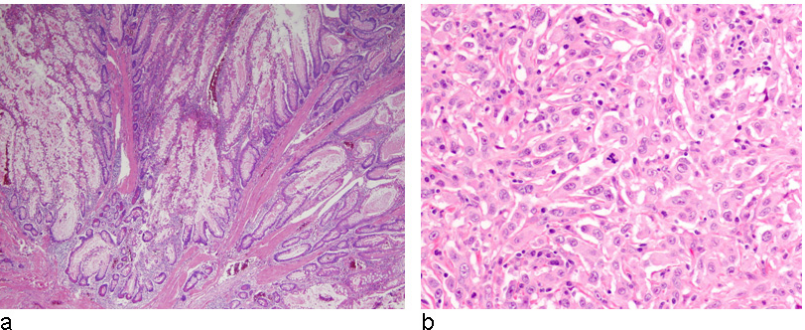
**Photomicrographs**. (a) Haematoxylin and Eosin stain (100×) of a hamartomatous polyp from the descending colon showed proliferation of dilated mucinous glands surrounded by smooth muscle fibers. This latter feature is characteristic of hamartomatous polyps observed in patients with Peutz-Jegher syndrome. (b) Photomicrograph of the left kidney tumor was composed of a solid proliferation of spindle cell with large irregular nucleus, abundant cytoplasm, and prominent mitotic figures. The findings are consistent with a high-grade clear cell renal cell carcinoma, sarcomatoid type.

At his initial NIH visit, the patient reported resection of multiple colonic polyps on successive colonoscopies, beginning in his early 50 s. Materials from several excised polyps were reviewed at the NIH Laboratory of Pathology, and revealed a mixed polyposis of 5 adenomatous polyps, 2 hamartomatous polyps (Figure 1a), and multiple hyperplastic polyps.

**Figure 2:**
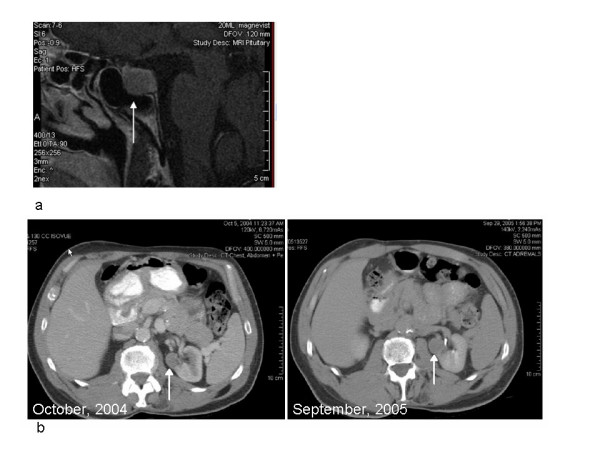
**Imaging studies**. (a) MRI of pituitary adenoma – T1 weighted sagittal MRI of the pituitary gland after intravenous contrast reveals an enlarged gland that is centrally hypoenhancing, with a rim of relative hyper-enhancement at the superior margin. The longest dimension of the pituitary is approximately 2 cm. (b) Composite of CT images of the left kidney, first visit and one year later- Serial contrast media-enhanced CTs of the abdomen demonstrate an enlarging left renal mass. The initial scan (October 2004) demonstrates a solid enhancing mass (white arrow) measuring approximately 2.6 cm. Follow-up CT approximately one year later shows slight enlargement (3.0 cm) (white arrow).

Physical examination revealed a muscular, mesomorphic white male (he is an avid body-builder) with multiple subcutaneous lipomas in the extremities and trunk. He had multiple dark brown to black, irregularly-shaped pigmented macules on the chest, back, arms, and legs, which were clinically suggestive of dysplastic nevi. Biopsy of the clinically most atypical lesion showed a simple lentigo, without melanocytic atypia. Protocol-related blood work, including testosterone, estradiol, chemistry panel, prostate specific antigen, alpha-fetoprotein, follicle stimulating hormone, luteinizing hormone, and beta-HCG, were normal. Ultrasound of the right testis showed early microlithiasis. Computed tomography of the chest, abdomen, and pelvis revealed an unexpected left inferomedial renal mass (Figure 2b), which was confirmed by MRI. Further evaluation of the left kidney mass was recommended, but not pursued due to other medical issues.

He returned for a scheduled clinic visit one year later and reported having an elevated growth hormone (GH) level detected during a complementary/alternative health evaluation. The patient also stated that his head and hands had been "getting bigger". He reported taking various dietary supplements, none of which was found to contain androgens upon review of their ingredients, as well as testosterone gel, which was started at the time of his complementary/alternative health evaluation due to a low serum testosterone level. On examination, he had an acromegalic appearance, including protruding supra-orbital ridges, a prognathic jaw, wide nose, and diffuse soft-tissue thickening of the hands, all of which were judged clinically to be more prominent when compared with the year before.

Laboratory studies demonstrated a normal fasting glucose, elevated levels of insulin-like growth factor-I (IGF-I) [943 ng/ml; normal range 71–290 ng/ml] and IGF binding protein-3 [8.4 μg/ml; normal range 3.2–6.6 μg/ml]. A pituitary MRI revealed a hypoenhancing, diffusely-enlarged pituitary measuring approximately 2 cm, consistent with an adenoma (Figure 2a). A follow-up renal CT showed the left inferomedial kidney mass had grown compared with the study performed one year earlier; the adrenal glands were normal.

On January 23, 2006, the patient underwent trans-sphenoidal resection of the pituitary mass, yielding a GH-producing adenoma. On February 22, 2006, he underwent a left nephrectomy for a stage III, poorly-differentiated renal cell carcinoma, with sarcomatoid features (Figure 1b). By November 2006, his growth hormone levels had returned to normal, and he had no evidence of recurrent kidney cancer.

Detailed family history review revealed a son with seminoma (confirmed by outside pathology report) at age 34 (cancer-free at age 36), and a sister who developed urinary bladder cancer (death certificate confirmation) at age 53. His son had no evidence of lipomatosis or multiple lentigines on examination at the time of study enrollment, and has had a negative screening colonoscopy. The proband's father was reported to have lipomatosis, but was deceased. There is no history of other benign or malignant neoplasms reported in the rest of the family.

Based on his personal history of multiple cancers, a pituitary adenoma, multiple pigmented skin lesions, multiple lipomas, and multiple colon polyps with varying histology, the patient underwent genetic testing for Cowden syndrome (the *PTEN *gene), Carney complex (the *PRKAR1A *gene), and multiple endocrine neoplasia syndrome type 1 (the *MEN1 *or *menin *gene). No germline mutations were detected in any of these genes.

## Discussion and conclusion

Our patient presented with multiple malignant and benign neoplasms including left testicular seminoma, left kidney renal cell carcinoma, a growth hormone-secreting pituitary adenoma, multiple pigmented skin lesions, lipomas, and colon polyps with varying histology (adenomatous, hyperplastic, and hamartomatous). The proband's son had testicular cancer and microlithiasis, but none of the other neoplasms, and his father is reported to have had lipomatosis. The clinical phenotype of the familial TGCT syndrome, as currently understood, is comprised of a site-specific predisposition to TGCT and does not appear to include an increased risk for other benign or malignant neoplasms [1,3]. Various aspects of testicular dysgenesis and genitourinary development have been implicated in the pathogenesis of familial TGCT, but this hypothesis remains unproven at present. As part of the protocol under which the current family was seen, we have evaluated 136 members of 35 multiple-case TGCT families, including 56 men with a history of TGCT; the constellation of abnormalities seen in the proband reported here is unique in our experience (Korde L, Kramer J, Mueller CM, and Greene MH, personal communication). Therefore, the possibility that the neoplasms observed in this patient represent unrecognized manifestations of the familial TGCT syndrome seems unlikely.

We considered whether these observations might be explained on the basis of a known genetic cancer susceptibility disorder and felt that Cowden syndrome, Carney complex, and multiple endocrine neoplasia type 1 warranted serious consideration in this regard, although none can account for all of the findings described herein (Table 1).

**Table 1:** Summary of patient's clinical findings relative to acromegaly and the three hereditary cancer susceptibility syndromes considered

Patient's Findings	Acromegaly	Hereditary Syndrome		
		
		Cowden syndrome	Carney complex	MEN1
Seminoma	**X**		XX*	
Pituitary adenoma (GH-producing)	**XX**		**XX**	**XX**
Colon adenomatous polyps	**XX**			
Colon harmatomatous polyps		**XX**		
Colon hyperplastic polyps				
Renal cell carcinoma	**X**	**X**		
Lipomas		**XX**		**XX**
Lentigines			**XX**	

XX: Classical syndrome-related findings

X: Findings are less clearly established as part of the syndrome

* Testicular cancers associated with Carney complex are typically large-cell calcifying Sertoli cell tumors or Leydig cells tumors of the testis

Cowden syndrome (CS), an autosomal dominant disease associated with mutations in the *PTEN *tumor suppressor gene on chromosome 10q10.22-23, is associated with elevated risks of breast and thyroid cancer, as well as diverse benign neoplasms including gastrointestinal hamartomatous polyps, lipomas, giant fibroadenomas of the breast, hemangiomas, and multiple early-onset uterine leiomyomas [5]. Kidney cancer and testicular neoplasm have been reported in patients with CS, but their association with this syndrome remains unproven. Our patient had multiple lipomas and colonic hamartomatous polyps, both of which are consistent with CS, but his renal cancer and pituitary adenoma cannot be readily explained on that basis. The patient's kidney cancer was located in an area of the kidney that is likely to have been included within the field of radiation delivered to the retroperitoneum (port films could not be located). Increased risk of second primary kidney cancer (RR = 2.8; 95% CI 2.1–3.8) among TGCT patients treated with post-operative radiation has been reported [4]. Therefore, the renal cancer could be a late complication of anti-cancer therapy, rather than a component of a genetic syndrome. The patient underwent commercial genetic testing for germline mutations in the *PTEN *gene, with no mutation detected. However, a mutation is identified by conventional testing in only 80% of patients who meet the CS clinical criteria, as they can have a large deletion or mutations in the promoter region of *PTEN*. Consequently, CS has not been completely excluded, but this diagnosis is unlikely since his family does not meet the clinical diagnostic criteria.

Carney complex (CNC) is a multiple neoplasia syndrome characterized by skin lesions (e.g., lentigines, compound nevi, and blue nevi), psammomatous melanotic schwannoma, cardiac myxomas, large-cell calcifying Sertoli cell tumors and Leydig cell tumors of the testis, as well as endocrine tumors, including primary pigmented nodular adrenocortical disease and pituitary adenoma, most of which are GH-producing [6,7]. CNC is associated with germline mutations in the *PRKAR1A *gene on chromosome 17q22-24, which are detected in 50–65% of CNC patients [6]. About 30% of CNC families have been linked to a susceptibility locus on chromosome 2p16 (CNC2), but the gene at this locus has not yet been identified. Our patient had a GH-producing pituitary adenoma and testicular cancer. However, CNC does not include colon polyps, lipomas or renal cancer among its recognized manifestations. He underwent testing for germline mutations in the *PRKAR1A *gene; no mutation was identified.

Multiple endocrine neoplasia type 1 (MEN1) involves tumors of the parathyroid, endocrine pancreas, anterior pituitary, adrenal glands, and carcinoid tumors of the GI tract. It is associated with germline mutations in the *MEN1 *gene, on chromosome 11q13 [8]. Anterior pituitary adenomas, about 1/4 of which are GH-producing, are found in 10%–60%, and multiple lipomas are found in about 30% of patients with MEN1 [8]. Both the patient and his father had multiple lipomas. The father was not known to have manifested other signs or symptoms suggestive of MEN1. The patient underwent germline mutation testing of the *MEN1 *gene, with no mutation detected.

*AIP *(which encodes for the aryl hydrocarbon receptor-interacting protein) has recently been implicated as a low-penetrance, familial pituitary adenoma susceptibility gene [9]. Although there are no other cases of pituitary adenoma in this family, it is not presently known whether other neoplasms occur excessively in pituitary adenoma kindred. We therefore tested the patient for mutations in *AIP; *none were detected (CA Stratakis, personal communication).

Finally, testicular germ cell tumor has been described in acromegalic patients [10]. Renal cancer, colon cancer, and colon polyps have been reported to occur excessively in acromegaly; however, the polyps observed in acromegalic patients are adenomatous or hyperplastic; hamartomatous polyps have not been described in this context [11,12]. Our patient's seminoma was unlikely to be related to acromegaly, given the temporal sequence of events, and neither lipomatosis nor lentigines are part of the acromegaly phenotype. However, we can not rule out the possibility of an association between acromegaly and either renal cancer and/or adenomatous and hyperplastic colon polyps in this patient.

While our patient had some of the clinical features associated with each of these cancer susceptibility disorders, none can account for his entire clinical phenotype, and germline mutation testing of the three genes implicated in the inherited diseases was negative. Conventional clinical mutation testing may have failed to detect a mutation in one of these genes; however, this likelihood is small since his family does not meet the clinical diagnostic criteria for any of these syndromes. He might represent an atypical presentation of some other known, multiple benign and malignant neoplasm susceptibility disorders (e.g., juvenile polyposis syndrome, Peutz-Jeghers syndrome, hereditary hemorrhagic telangiectasia, or *MYH*-adenomatous polyposis); research evaluation of a wider spectrum of genes is now underway. Or, he may have several co-existing but etiologically-unrelated conditions, e.g., familial testicular cancer; acromegaly with renal cancer and colon polyps; and idiopathic lipomatosis.

The final possibility is that this family's phenotype represents a novel neoplasm susceptibility disorder, of which TGCT is an important component. Of course, this hypothesis cannot be tested on the basis of a single case report. We await with interest future observations from other clinical investigators, in response to the report we have provided herein.

## Competing interests

The author(s) declare that they have no competing interests.

## Authors' contributions

PM had primary responsibility for drafting the manuscript. LK, JK, and MHG were responsible for the design and operation of the Familial Testicular Cancer Study, contributed to this patient's evaluation, and contributed to writing the manuscript. JP, CM, and SP were involved in this patient's evaluation, genetic counseling, and data collection and medical record abstraction. CS evaluated the patient for acromegaly and performed genetic testing for Carney complex, MEN 1, and familial pituitary adenoma. PP, WML, GB, MM, and PC were involved in the patient's clinical assessment and treatment. All authors read and approved the final manuscript

## References

[B1] Holzik MFL Rapley EA Hoekstra HJ Sleijfer DT Nolte IM Sijmons RH Genetic predisposition to testicular germ-cell tumours Lancet Oncol 2004 5 6 363 371 10.1016/S1470-2045(04)01493-7 15172357

[B2] Crockford GP Linger R Hockley S Dudakia D Johnson L Huddart R Tucker K Friedlander M Phillips KA Hogg D Jewett MAS Lohynska R Daugaard G Richard S Chompret A Bonaiti-Pellie C Heidenreich A Albers P Olah E Geczi L Bodrogi I Ormiston WJ Daly PA Guilford P Fossa SD Heimdal K Tjulandin SA Liubchenko L Stoll H Weber W Forman D Oliver T Einhorn L McMaster M Kramer J Greene MH Weber BL Nathanson KL Cortessis V Easton DF Bishop DT Stratton MR Rapley EA Genome-wide linkage screen for testicular germ cell tumour susceptibility loci Hum Mol Genet 2006 15 3 443 451 10.1093/hmg/ddi459 16407372

[B3] Holzik MFL Sijmons RH Sleijfer DT Sonneveld DJA Hoekstra-Weebers JEHM van Echten-Arends J Hoekstra HJ Syndromic aspects of testicular carcinoma Cancer 2003 97 4 984 992 10.1002/cncr.11155 12569597

[B4] Travis LB Fossa SD Schonfeld SJ McMaster ML Lynch CF Storm H Hall P Holowaty E Andersen A Pukkala E Andersson M Kaijser M Gospodarowicz M Joensuu T Cohen RJ Boice JD Jr. Dores GM Gilbert ES Second cancers among 40 576 testicular cancer patients: focus on long-term survivors J Natl Cancer Inst 2005 97 18 1354 1365 16174857 10.1093/jnci/dji278

[B5] Schreibman IR Baker M Amos C McGarrity TJ The Hamartomatous Polyposis Syndromes: A clinical and molecular review Am J Gastroenterol 2005 100 2 476 490 10.1111/j.1572-0241.2005.40237.x 15667510

[B6] Stratakis CA Kirschner LS Carney JA Clinical and molecular features of the Carney Complex: Diagnostic criteria and recommendations for patient evaluation J Clin Endocrinol Metab 2001 86 9 4041 4046 10.1210/jc.86.9.4041 11549623

[B7] Watson JC Stratakis CA Bryant-Greenwood PK Koch CA Kirschner LS Nguyen T Carney JA Oldfield EH Neurosurgical implications of Carney complex Journal of Neurosurgery 2000 92 3 413 418 10701527 10.3171/jns.2000.92.3.0413

[B8] Brandi ML Gagel RF Angeli A Bilezikian JP Beck-Peccoz P Bordi C Conte-Devolx B Falchetti A Gheri RG Libroia A Lips CJM Lombardi G Mannelli M Pacini F Ponder BAJ Raue F Skogseid B Tamburrano G Thakker RV Thompson NW Tomassetti P Tonelli F Wells SA Jr. Marx SJ Guidelines for diagnosis and therapy of MEN type 1 and type 2 J Clin Endocrinol Metab 2001 86 12 5658 5671 10.1210/jc.86.12.5658 11739416

[B9] Vierimaa O Georgitsi M Lehtonen R Vahteristo P Kokko A Raitila A Tuppurainen K Ebeling TML Salmela PI Paschke R Gundogdu S De Menis E Makinen MJ Launonen V Karhu A Aaltonen LA Pituitary adenoma predisposition caused by germline mutations in the AIP gene Science 2006 312 5777 1228 1230 10.1126/science.1126100 16728643

[B10] Abraham Couldwell Bilateral testicular enlargement and seminoma in a patient with acromegaly Br J Neurosurg 2004 18 6 629 631 10.1080/02688690400022797 15799199

[B11] Jenkins PJ Acromegaly and cancer Horm Res 2004 62 Suppl 1 108 115 10.1159/000080768 15761242

[B12] Martino A Cammarota G Cianci R Bianchi A Sacco E Tilaro L Marzetti E Certo M Pirozzi G Fedeli P Pandolfi F Pontecorvi A Gasbarrini G Marinis LD High prevalence of hyperplastic colonic polyps in acromegalic subjects Dig Dis Sci 2004 49 4 662 666 10.1023/B:DDAS.0000026315.91800.b2 15185875

